# Study on the Mechanism and Dose–Effect Relationship of Flavonoids in Different Extracts of Radix Hedysari Against Gastrointestinal Injury Induced by Chemotherapy

**DOI:** 10.3390/ph18071072

**Published:** 2025-07-20

**Authors:** Shasha Zhao, Miaomiao Yang, Zimu Yang, Hai He, Ziyang Wang, Xinyu Zhu, Zhijia Cui, Jing Shao

**Affiliations:** 1 College of Pharmacy, Gansu University of Traditional Chinese Medicine, Lanzhou 730000, China; zhaoshasha1112@163.com (S.Z.); 15294350715@163.com (M.Y.); qitian_122@163.com (Z.Y.); hehai1128@126.com (H.H.); yingtao20@126.com (Z.W.); zhxy19@gszy.edu.cn (X.Z.); zhijiacui@126.com (Z.C.); 2 State Administration of Traditional Chinese Medicine Class III Laboratory (Key Laboratory of Traditional Chinese Medicine Chemistry), Lanzhou 730000, China; 3 Northwest China–Tibet Medicine Collaborative Innovation Center, Lanzhou 730000, China

**Keywords:** Radix Hedysari, chemotherapy, gastrointestinal injury, mechanism of action, flavonoids, correlation

## Abstract

**Background:** Previous studies have shown Radix Hedysari (RH)’s gastroprotective potential, but its active components and mechanisms remain uncharacterized. This study aimed to identify RH’s bioactive fractions, elucidate protection mechanisms, establish flavonoid dose-effect relationships, and determine the pharmacodynamic basis. **Methods**: Chemical profiling quantified eight flavonoids via HPLC. Network pharmacology screened targets/pathways using TCMSP, GeneCards databases. In vivo validation employed cisplatin–induced injury models in Wistar rats (n = 10/group). Assessments included: behavioral monitoring; organ indices; ELISA (MTL, VIP, IFN–γ, IgG, IL–6, TNF–α etc.); H&E; and Western blot:(SCF, c–Kit, p65). Dose–effect correlations were analyzed by PLS–DA. **Results**: Content determination indicated that Calycosin–7–glucoside and Ononin were notably enriched on both the n–BuOH part and the EtOAc part. Network pharmacology identified 5 core flavonoids and 8 targets enriched in IL–17/TNF signaling pathways. n–BuOH treatment minimized weight loss vs. MCG, increased spleen/thymus indices. n–BuOH and HPS normalized gastrointestinal, immune, inflammatory biomarkers (*p* < 0.01 vs. MCG). Histopathology confirmed superior mucosal protection in n–BuOH group vs. MCG. Western blot revealed n–BuOH significantly downregulated SCF, c–kit, and p65 expressions in both gastric and intestinal tissues (*p* < 0.001 vs. MCG). PLS–DA demonstrated Calycosin–7–glucoside had the strongest dose–effect correlation (VIP > 1) with protective outcomes. **Conclusions:** The n–BuOH fraction of RH is the primary bioactive component against chemotherapy–induced gastrointestinal injury, with Calycosin–7–glucoside as its key effector. Protection is mediated through SCF/c–Kit/NF–κB pathway inhibition, demonstrating significant dose–dependent efficacy. These findings support RH’s potential as a complementary therapy during chemotherapy.

## 1. Introduction

Chemotherapy is one of the main methods of comprehensive tumor cell suppression and is the cornerstone of current cancer treatment [1]. Chemotherapy is highly efficacious but inevitably toxic to normal tissues and organs, leading to complications such as myelosuppression, gastrointestinal toxicity [2], and decreased immune function [3]. Cisplatin is a commonly used platinum–based chemotherapeutic agent with non–specific cytotoxicity, which inhibits the DNA replication process and disrupts the cell membrane structure of cancer cells. However, cisplatin can cause nephrotoxicity and gastrointestinal damage, nausea, vomiting, and diarrhea [4]. It is critical to use cisplatin to suppress tumor cells while minimizing its damage to normal tissue cells.

“Zhenqi Fuzheng Granules” are a commonly used proprietary Chinese medicine, often used in conjunction with chemotherapy to alleviate the side effects of chemotherapy and to promote the recovery of normal functions [5]. RH is a traditional Chinese medicine and edible plant extracted from the dried root of Hedysarum polybotrys Hand.–Mazz, which has anti–tumor activity [6] and confers protection of gastric mucosa [7,8]. Radix Astragali (RA) is one of the main ingredients of “Zhenqi Fu Zheng Granules”, and RH has been used under Astragalus since ancient times, which confirms its medicinal status. RH and RA are efficacious in the regulation of gastrointestinal and immune functions in splenic deficiency syndrome, showing significant differences in the regulation of the immune damage factors, such as IL–6, IFN–γ, TNF–α, and IgM in serum, as well as gastrointestinal damage factors such as D–xylose, MTL, and GAS, and the efficacy of RH was superior to that of RA [9]. Additionally, RH can be used as a functional food to alleviate ulcerative colitis (UC) induced by 2,4–Dinitrobenzenesulfonic acid [10]. Based on these findings, consequently, we speculate that RH, akin to RA, may represent a promising functional food for mitigating gastrointestinal damage induced by chemotherapy. Despite the clear gastrointestinal protective effect of RH, the mechanism and material basis of its protective effect against chemotherapy–induced gastrointestinal injury are still unclear.

Studies had shown that flavonoids, as a chemopreventive agent for cancer, were mainly absorbed, distributed, and metabolized in the gastrointestinal tract [11,12]. Another study found that flavonoids can regulate gastrointestinal function [13]. Flavonoids, as one of the main active parts of RH, have been found to inhibit the process of pulmonary fibrosis [14] and also had anti–tumor effects [15].

**Figure 1:**
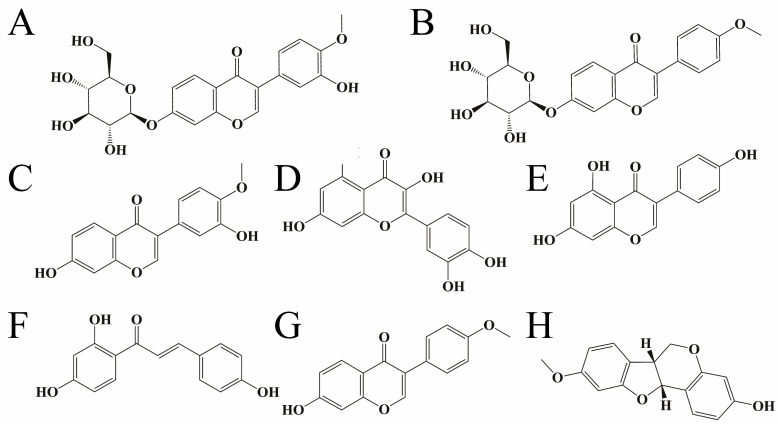
Structural formulae of eight flavonoid compounds ((**A**) Calycosin–7–glucoside; (**B**) Ononin; (**C**) Calycosin; (**D**) Quercetin; (**E**) Genistein; (**F**) Isoliquiritigenin; (**G**) Formononetin; (**H**) Medicarpin).

Our group had conducted basic research on the immunomodulatory [16] and cardio–protective effects [17] of RH and screened the dose of RH for its protective effects in a high–dose [18] group. In this study, we also screened the active parts and principal components of RH flavonoids for their protective and anti–tumor effects on organs. We comprehensively analyzed the content and activity indicators of eight main components (Figure 1) to explore the “dose–effect” relationship of the flavonoid active components of RH in combating gastrointestinal damage caused by chemotherapy. We further verified and explored the potential molecular mechanism. To study the correlation between the small molecules of RH and gastrointestinal protection induced by chemotherapy at a microscopic level, and to find out its mechanism of action.

## 2. Results

### 2.1. Determination of Principal Components in Different Extracts

The results are presented in Table 1, where the content of Calycosin–7–glucoside in the WE and n–BuOH groups was the highest, reaching 3.1049 μg/g and 6.8075 μg/g. Meanwhile, the highest content of Ononin in the 95% EtOH and EtOAc groups was 2.8848 μg/g and 22.2778 μg/g, respectively. Additionally, the highest content of Calycosin in the CHCl_3_ part was 2.0956 μg/g, while the highest content of Medicarpin in PE was 0.3220 μg/g. Lastly, RWE exhibited a higher Genistein content of 0.5757 μg/g.

### 2.2. Results of the Network Pharmacology Analysis

#### 2.2.1. Component Screening and Target Prediction

Nine flavonoids were retrieved from the TCMSP database. In addition, considering the possible limitations of the database, in order to avoid the omission of the screening of potential active components,4 components, such as Genistein, Calycosin–7–glucoside, Isoliquiritigenin, and Liquiritigenin, which were determined in 2.1, were added, totaling 13 flavonoids. A total of 168 targets of each component were obtained on the platforms of TCMSP and Swiss Target Prediction.

#### 2.2.2. Acquisition of Disease Targets and Their Intersection Mapping with Component Targets

**Figure 2:**
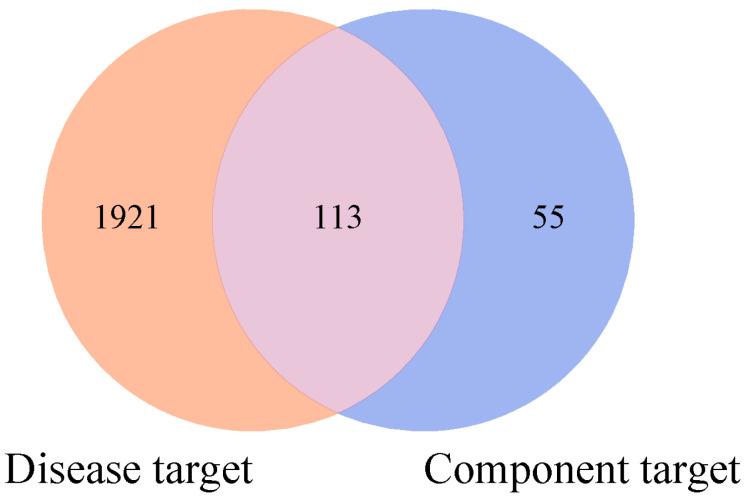
Venn diagram of the predictive targets of RH against gastrointestinal injury caused by chemotherapy. (Areas in yellow represent chemotherapy–induced gastrointestinal injury targets (2034), blue represent RH component targets (168), and purple indicates overlapping targets (113)).

A total of 2034 predicted targets of “gastrointestinal injury caused by chemotherapy” were obtained from the Genecards, CTD, and OMIM databases, and 113 potential targets were obtained by mapping and the intersection of component targets and disease targets on the Draw Venn Diagram platform, as shown in Figure 2.

#### 2.2.3. Construction of “Drug–Component–Target” Network

**Figure 3:**
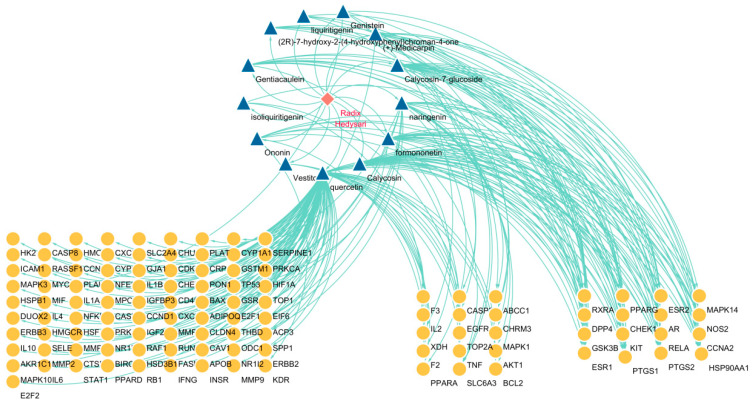
Diagram of “Herbs–Components–Targets”. (Quercetin, Calycosin, Formononetin, Naringenin, and Calycosin–7–glucoside were used as key compounds. The red diamond represents the drug RH, the blue triangle represents the compound, and the yellow circle represents the target.).

The 13 components and 113 targets obtained in Section 2.2.2 were imported into Cytoscape 3.10.2 software to construct a visual network, as shown in Figure 3. Targets are divided into three categories, which represent, from left to right, that they can be regulated by 1 compound (82), 2–3 compounds (15), and more than 4 compounds (16). Taking the average value of their degrees, there are four compounds greater than 18, namely Quercetin (95), Calycosin (29), Formononetin (20), Naringenin (20), and Calycosin–7–glucoside (19), which can be used as key compounds.

#### 2.2.4. Construction of PPI Protein Interaction Network and Screening of Key Targets

**Figure 4:**
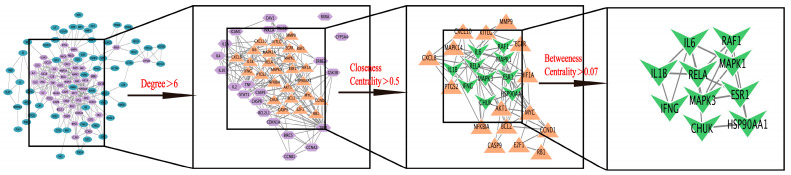
PPI network screened by topological features. (IL6, SP90AA1, MAPK3, MAPK1, ESR1, IL1B, IFNG, and RELA were used as key targets. Node attributes: blue circles (all predicted targets), pentagons (Degree Centrality > 6), triangles (Closeness Centrality > 0.5), V–shapes (Betweenness Centrality > 0.07)).

In total, 113 genes from Section 2.2.2 were imported into the String database to obtain the primary interaction network. The Degree Centrality, Closeness Centrality, and Betweenness Centrality of its topological structures were analyzed using Cytoscape 3.10.2 software. The average values were taken to make them all greater than the average value, and eight key targets in the network of RH’s anti–gastrointestinal injury induced by chemotherapy were screened: IL6, HSP90AA1, MAPK3, MAPK1, ESR1, IL1B, IFNG, and RELA. The PPI network diagram was shown in Figure 4, which was screened according to different conditions. The target information is shown in Table 2.

#### 2.2.5. GO Analysis

**Figure 5:**
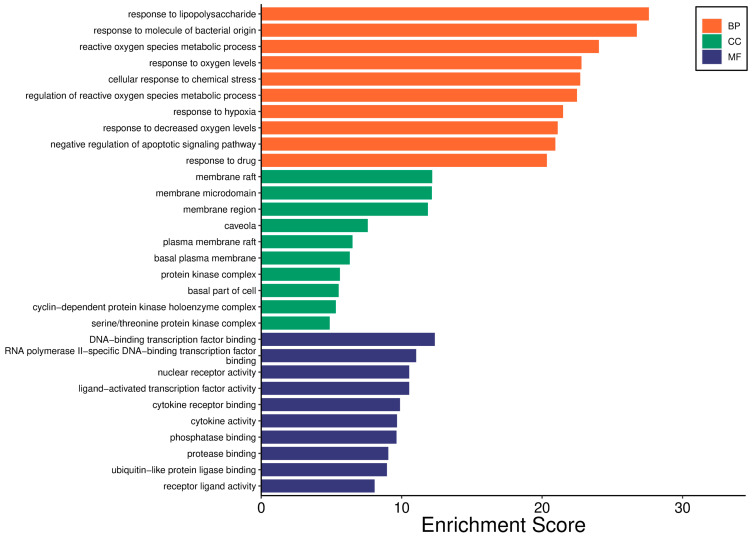
GO enrichment analysis of RH targets against chemotherapy–induced gastrointestinal injury. The top 10 significant terms (*p* < 0.01) are displayed for Cellular Components (CCs, e.g., membrane rafts), Biological Processes (BPs, e.g., chemical stress response), and Molecular Functions (MFs, e.g., transcription factor binding). The results reveal RH’s multi–level regulatory roles in membrane organization, oxidative stress modulation, and transcriptional control.

In total, 113 targets screened in Section 2.2.2 were introduced into the micro–information platform for GO analysis, and the top 10 targets were displayed, as shown in Figure 5. GO analysis revealed (*p* value < 0.01) 46 Cellular Components (CCs). The target genes of RH against gastrointestinal injury induced by chemotherapy were most significantly related to the membrane raft, membrane microdomain, plasma membrane raft, basal plasma membrane, protein kinase complex, cyclin–dependent protein, serine/three–one protein kinase complex, etc. Genes were involved in 1835 Biological Processes (BPs), mainly enriched to the following categories: cellular response to chemical stress, regulation of reactive oxygen species metallic process, response to drug, reactive oxygen species metallic process, response to degraded oxygen levels, etc. Genes were enriched in 116 Molecular Function (MFs), such as DNA–binding transcription factor binding, RNA polymerase II–specific DNA–binding transcription factor binding, nuclear receptor activity, ligand–activated transcription factor activity, cytochrome receptor binding, and receptor ligand activity.

#### 2.2.6. KEGG Pathway Analysis

**Figure 6:**
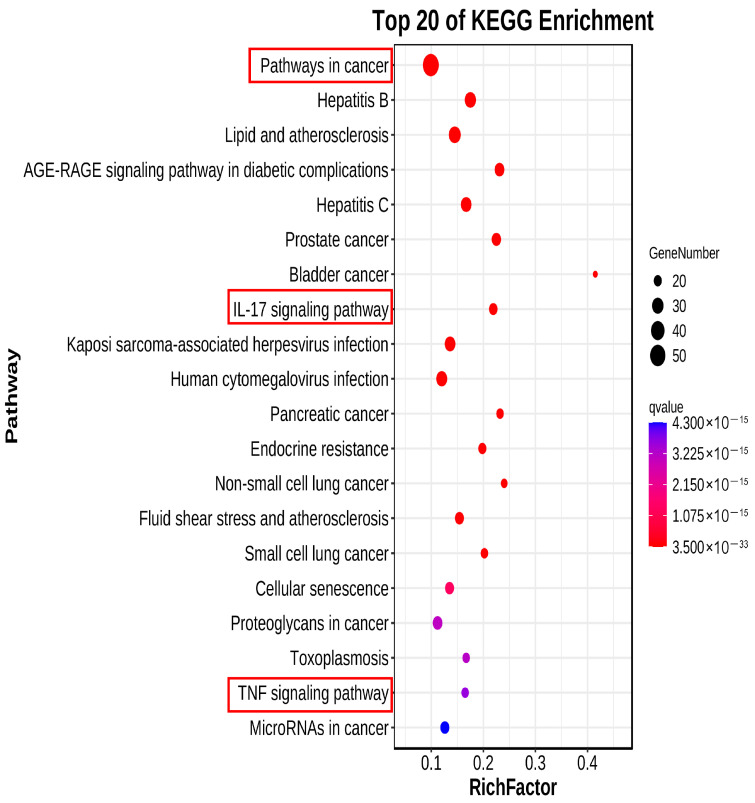
The first 20 enriched KEGG pathways of RH against chemotherapy–induced gastrointestinal injury. The pathway in cancer shows the highest Gene Number and the lowest Q value, indicating its leading role in immune/inflammatory regulation. IL–17 and TNF signaling pathways are also important immune and inflammatory pathways. The dot size reflects the number of genes, while the intensity of color indicates importance.

A total of 259 pathways were enriched by KEGG analysis (*p* value < 0.01), and the top 20 significantly different pathways are shown in Figure 6. In the first classification of Human Diseases, the pathways in cancer have the largest Richness Factor value and the smallest *p* value, which was the most important pathway in the anti–gastrointestinal injury caused by chemotherapy of RH. In addition, the IL–17 signaling pathway in the first classification of Organismal Systems and the TNF signaling pathway in the first classification of Environmental Information Processing were also important.

### 2.3. Screening of Effective Fractions and Study on Mechanism of Action

#### 2.3.1. General Behavioral Monitoring of Rats

**Figure 7:**
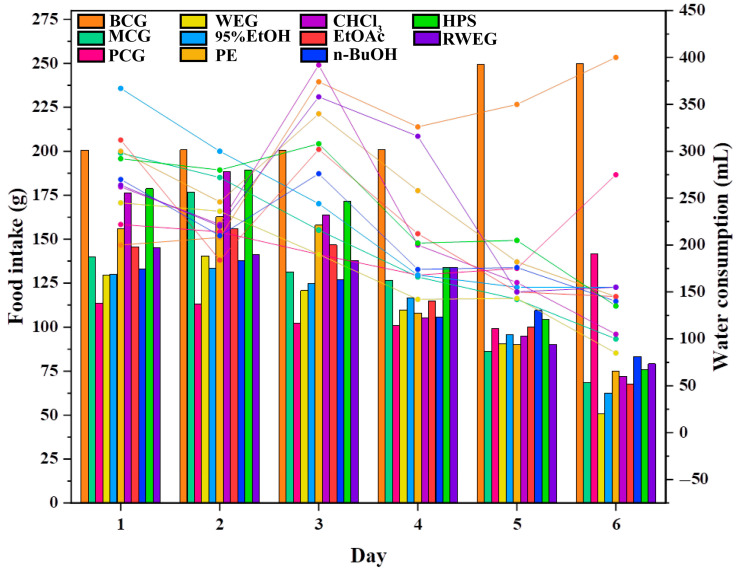
Changes in food intake and water consumption across experimental groups. The bar chart shows food intake, and the line chart shows water consumption. The BCG shows progressive increases in both parameters, while the MCG exhibits sustained declines, confirming successful cisplatin–induced gastrointestinal injury modeling. Notably, the n–BuOH group demonstrates partial recovery (increased food intake by day 5), suggesting its protective effect against chemotherapy–induced anorexia and dehydration.

**Figure 8:**
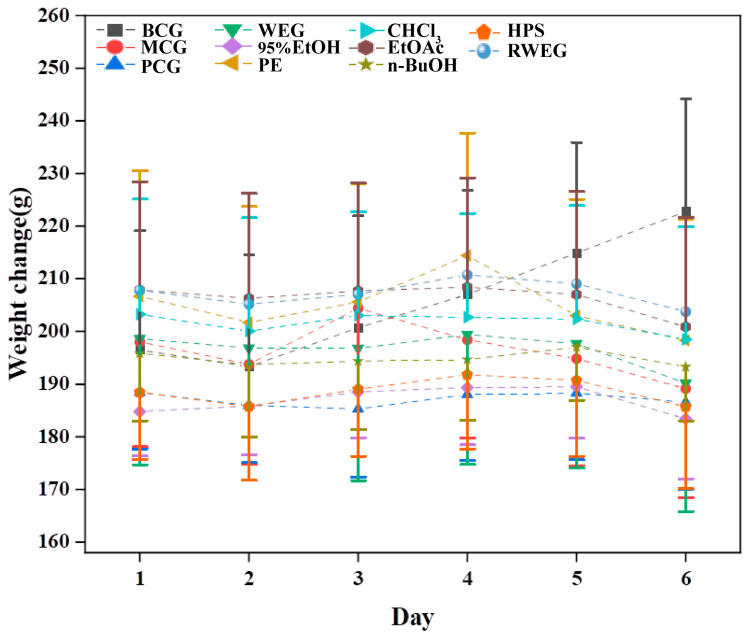
Body weight dynamics during chemotherapy intervention. While BCG rats gained weight steadily, MCG rats show progressive weight loss, validating the disease model. Drug–treated groups (e.g., n–BuOH) attenuated weight loss compared to MCG, with n–BuOH exhibiting the mildest reduction, consistent with its gastrointestinal protective role.

The changes in water consumption, food intake, and body weight of rats are shown in Figure 7 and Figure 8. The BCG exhibited a continuous increase in both food and water consumption, accompanied by a steady rise in body weight during the monitoring time. Compared with the BCG, the consumption of food and water in the MCG continued to decrease, and the weight also continued to decrease. In the PCG, the food intake, water intake and body weight decreased first and then increased after cisplatin modeling. Each drug group demonstrated a trend of initial weight gain followed by a decrease post–cisplatin modeling, alongside a continued decline in food intake and water consumption. Among them, the food intake of n–BuOH increased on the 5th after a continuous decrease, and the body weight decreased less within seven days compared with other groups.

As shown in Table 3, compared with the BCG, the spleen index and thymus index MCG had different shoulder letters, which had a very significant difference. In the spleen index drug group, compared with the MCG, the n–BuOH and the PE groups had different shoulder letters and heavier weights, and both parts had better protective effects on the spleen. As for the thymus index, compared with the MCG, the n–BuOH group was heavier weighted, and it had a better protective effect on the thymus.

#### 2.3.2. Detection of Serum Factors in Rats

**Figure 9:**
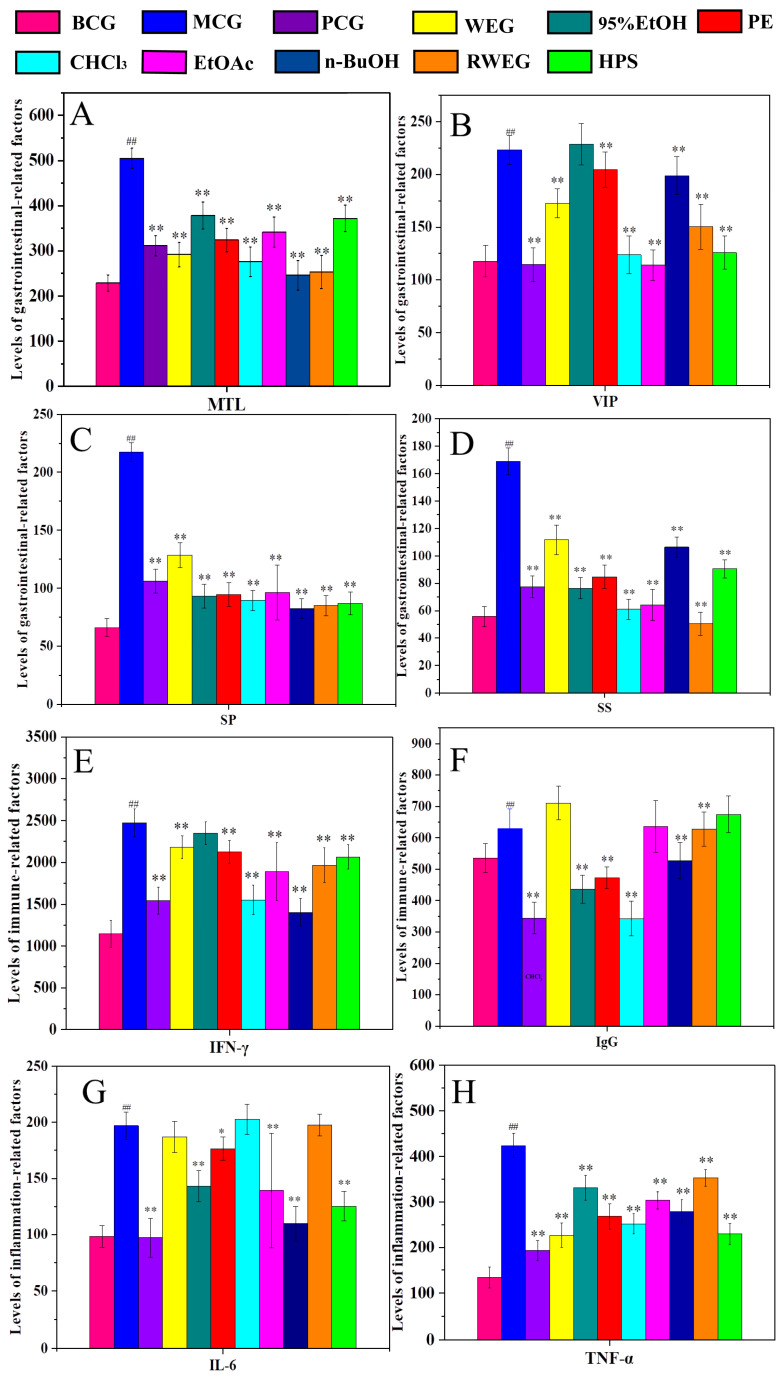
Serum biomarker levels. (**A**) Motilin (MTL); (**B**) Vasoactive intestinal peptide (VIP); (**C**) Somatostatin (SS); (**D**) Substance P (SP); (**E**) Interferon gamma (IFN–γ); (**F**) Immunoglobulin G (IgG); (**G**) Interleukin–6 (IL–6); (**H**) Tumor necrosis factor alpha (TNF–α). MCG significantly altered all measured biomarkers vs. BCG (*p* < 0.01). n–BuOH treatment showed near–normalization of MTL, VIP, and inflammatory cytokines. *^##^ p* < 0.01 vs. BCG; * p < 0.05 and ** p < 0.01 vs. MCG.

To evaluate the therapeutic and protective effects of different polar parts on rat gastrointestinal injury, systemic immune reduction, and inflammatory response, we measured the levels of gastrointestinal representative factors (MTL, VIP, SS, and SP) and immunoregulatory factors (IFN–γ and IgG) and inflammatory factors (IL–6 and TNF–α), as shown in Figure 9. We established the model by intraperitoneal injection of cisplatin and found that the levels of each factor in the MCG serum significantly increased or decreased compared to the BCG (*p* < 0.01). However, the levels of each factor in the PCG were significantly restored compared to the MCG (*p* < 0.01). The gastrointestinal representative factors MTL, SS, and SP showed significant differences in each drug group (*p* < 0.01), and the levels of VIP factors were significantly different from those in the MCG except for the 95% EtOH group (*p* < 0.01). The levels of IFN–γ were significantly different from those of the MCG except the 95% EtOH (*p* < 0.01), and the levels of IgG, except for EtOAc, RWEG, and HPS, were significantly different from those of the MCG. The content of TNF–α in each drug group was significantly different from that of the MCG (*p* < 0.01), and the level of IL–6 was significantly different from that of the MCG except WEG, CHCl_3_, and RWEG (*p* < 0.05 and *p* < 0.01).

#### 2.3.3. Histopathological Observation

##### H&E Staining of Gastric Antrum

**Figure 10:**
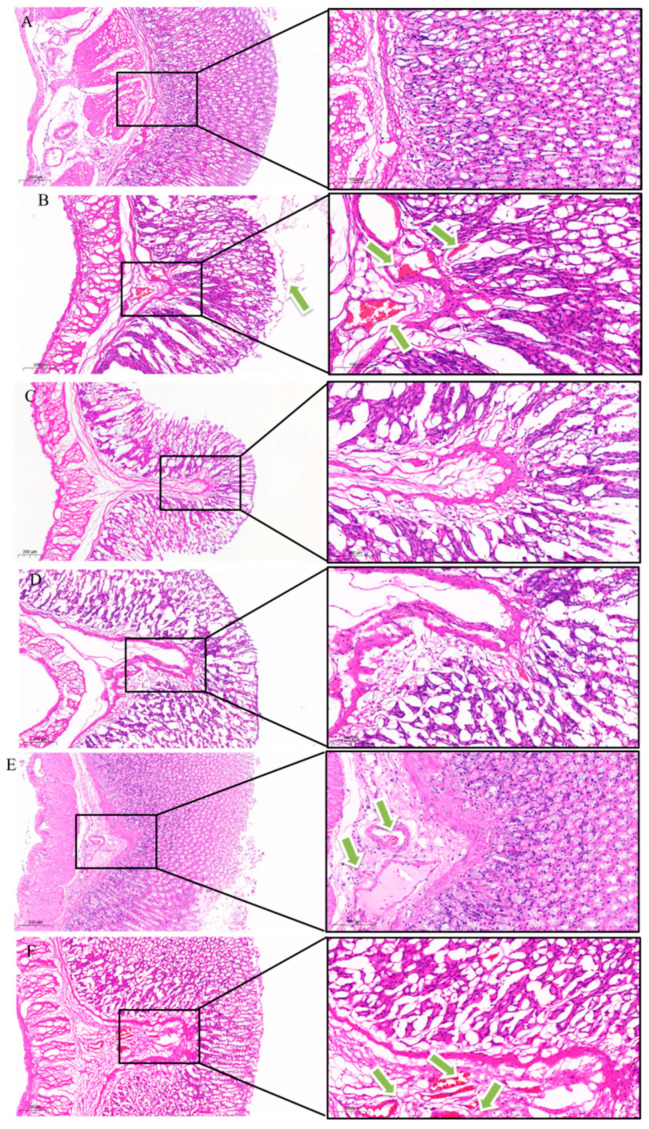
Histopathological evaluation of gastric tissues by H&E staining. (**A**) BCG, (**B**) MCG, (**C**) PCG, (**D**) n–BuOH, (**E**) HPS, and (**F**) CHCl_3_; left: 12×; right: 36×. The green arrows indicate the bleeding position. BCG shows intact gastric architecture with normal glands and no inflammation, while MCG exhibits severe mucosal necrosis, hemorrhage, and epithelial sloughing (arrows). Treatment groups demonstrate varying protection, with n–BuOH showing near–normal mucosal structure and minimal bleeding compared to MCG.

H&E staining of rat stomachs under a light microscope is shown in Figure 10. The BCG is shown in Figure 10A, with a clear and complete stomach structure, no gland damage, normal structure, and no inflammatory cell infiltration. In contrast, the stomach of the MCG shown in Figure 10B displays degeneration, necrosis, and shedding of the mucosal epithelial tissue. Notably, superficial mucosal cell necrosis was observed, with necrotic cells dissolving and leaving only outlines or undergoing shrinkage, accompanied by varying degrees of bleeding. Additionally, the submucosal connective tissue showed signs of bleeding. Compared with the MCG, the damage degree of the gastric mucosal tissue structure in the drug group was reduced. For Figure 10C, in the PCG, the structure of each layer of stomach is clear, the glands in the lamina propria are arranged neatly, and there is no cell congestion. Compared with the MCG, the damage degree of gastric mucosa structure in the drug group was less, and the damage of the n–BuOH mucosa layer was lighter, with fewer necrotic cells and micro–bleeding. In the HPS group, the mucosal epithelial tissue was incomplete, and the submucosal connective tissue was accompanied by a small amount of bleeding. CHCl_3_ showed infiltration of some cells in the lamina propria, degeneration and necrosis of the muscularis mucosa, and bleeding of connective tissue in the submucosa.

##### H&E Staining of Ileum

**Figure 11:**
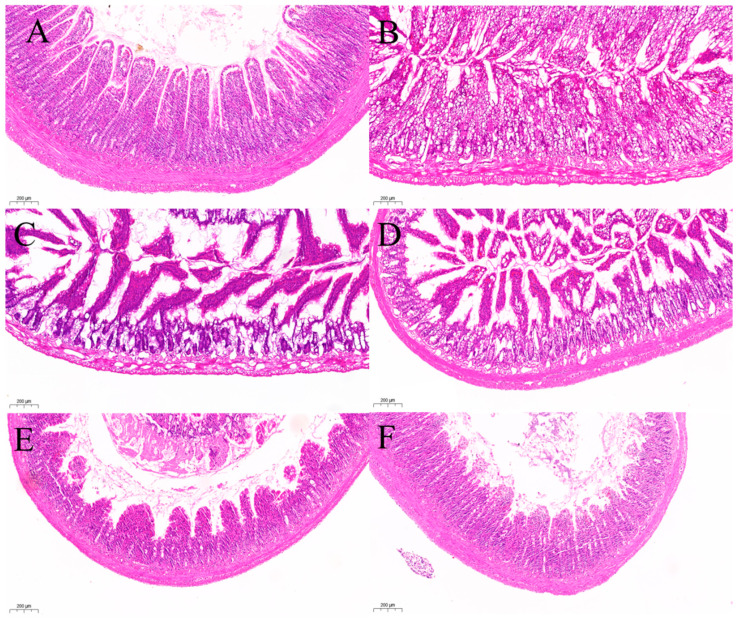
Histopathological evaluation of intestinal tissues by H&E staining. (**A**) BCG, (**B**) MCG, (**C**) PCG, (**D**) n–BuOH, (**E**) HPS, and (**F**) CHCl_3_. BCG exhibits intact intestinal villi with well–defined crypt structures, while MCG shows severe villous atrophy and crypt destruction, confirming a successful cisplatin–induced intestinal injury model. The n–BuOH treatment group demonstrates the most significant protective effect, with near–normal villous architecture and visible crypt regeneration, superior to the HPS and CHCl_3_ groups, which show partial mucosal recovery.

**Figure 12:**
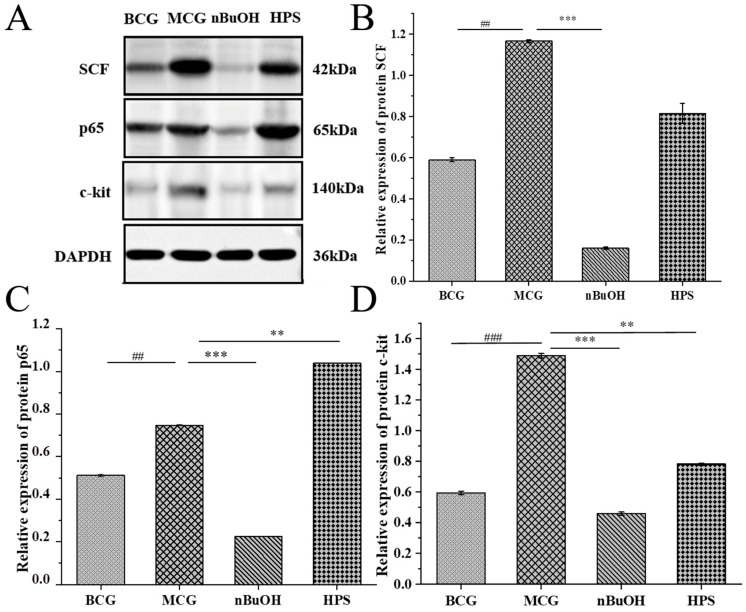
Western Blot analysis of gastric SCF/c–kit/p65 signaling pathway (*n* = 3) (Note: ## *p* < 0.01 and ### *p* < 0.001 compared with the BCG; compared with the MCG, ** *p* < 0.01 and *** *p* < 0.001). (**A**) Protein bands show cisplatin–induced up–regulation of SCF, c–kit, and p65 in MCG versus BCG (*p* < 0.01). (**B**–**D**) n–BuOH treatment significantly reversed these effects (*p* < 0.001), while HPS partially down–regulated c–kit (*p* < 0.01), confirming RH’s regulation of the SCF/c–kit/NF–κB pathway in gastric protection.

Figure 11 presents H&E staining of the intestines of rats from each group, as observed under a light microscope. The results indicate that the intestinal villous epithelium of the BCG, as shown in Figure 11A, was intact, abundant, and tightly arranged, with clearly defined crypts and a complete muscle layer. In contrast, Figure 11B illustrates that the intestinal villi in the MCG were damaged, with the crypt structure nearly absent and the intestinal gland structure completely lost. Figure 12C shows that the villi in the PCG were well organized and closely packed, with visible and distinct crypts; however, a small number of intestinal villi were separated from the lamina propria. Figure 11D reveals that the n–BuOH group exhibited more intact intestinal villi compared to the MCG, with visible crypts. In Figure 11E, the HPS demonstrates more complete intestinal villi than the MCG, with the crypt structure partially visible, although the mucosal layer was thinner than that observed in the n–BuOH. Finally, Figure 11F indicates that the villi in the CHCl_3_ were only partially visible, with the crypt structure nearly absent.

#### 2.3.4. Detection of Signal Pathway Protein Molecules

Observations of the general behavioral status of the rats, along with pathological section analyses and serum factor detection results, indicated significant medicinal effects from the n–BuOH and HPS groups. Consequently, these two groups were selected for Western blotting (WB) to assess the expression of stem cell factors (SCFs), tyrosine kinase receptors (c–kit), and NF–κB p65 protein in the cells of each group, facilitating research into the associated signaling pathways.

##### Molecular Detection of Gastric Protein

The WB detection results of gastric protein molecules is shown in Figure 12. Figure 12A presents the immunoblot electrophoresis bands of proteins c–kit, SCF, and p65 in the gastric antrum tissue of rats from each group, while Figure 12B–D depict the expression levels of proteins c–kit, SCF, and p65 in each the group. Compared with the BCG, the expression levels of proteins SCF, c–kit, and p65 in the MCG were significantly up–regulated *(p* < 0.01 or *p* < 0.001). At the same time, when compared to the MCG, the SCF, c–kit, and p65 protein expressions in n–BuOH were significantly down–regulated (*p* < 0.001). The expression of SCF in the HPS group was also down–regulated, although this change was not statistically significant, while the protein c–kit was significantly down–regulated (*p* < 0.01).

##### Molecular Detection of Intestinal Protein

**Figure 13:**
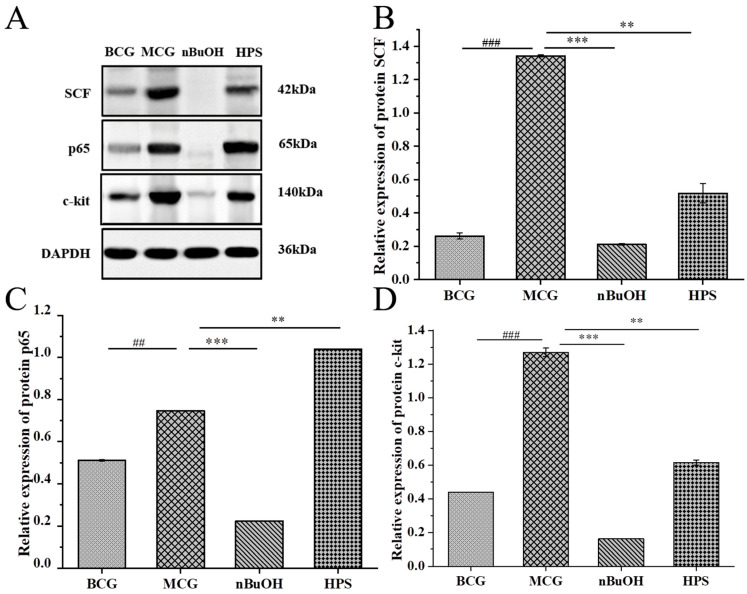
Western Blot analysis of intestinal SCF/c–kit/p65 signaling pathway (*n* = 3) (Note: ## *p* < 0.01 and ### *p* < 0.001 compared with the BCG; compared with the MCG, ** *p* < 0.01 and *** *p* < 0.001). (**A**) Protein bands demonstrate cisplatin–induced up–regulation of SCF, c–kit, and p65 in MCG versus BCG (*p* < 0.01). (**B**–**D**) n–BuOH treatment significantly reversed these effects (*p* < 0.001), while HPS down–regulated SCF and c–kit (*p* < 0.01), confirming RH’s regulation of this pathway in intestinal protection.

The results of WB detection of intestinal protein molecules are presented in Figure 13. The protein expressions of SCF, c–kit, and p65 in the MCG were significantly up–regulated (*p* < 0.01 or *p* < 0.001) compared to the BCG. Additionally, the expression and content of SCF, c–kit, and p65 protein were significantly down–regulated (*p* < 0.001) in comparison to the MCG, and the SCF and c–kit of the HPS group were significantly down–regulated (*p* < 0.01).

### 2.4. Correlation Analysis of “Dose–Effect”

The PLS–DA model was used to analyze the “dose–effect” relationship between eight active components and organ indexes in different extracts and serum factors related to gastrointestinal motility (MTL, VIP, SP, SS), immunity markers (IFN–γ, IgG), and inflammatory factors (IL–6, TNF–α), as detailed in Table 4.

#### 2.4.1. Correlation Analysis Between Organ Index and Main Ingredients

**Figure 14:**
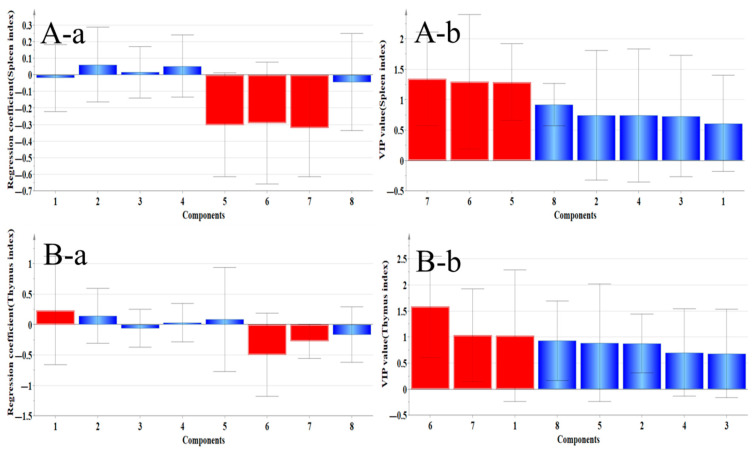
Correlation analysis between flavonoid components and immune organ indices. (**A**) While Ononin, Quercetin, and Calycosin show positive regression coefficients with the spleen index (**A-a**), their VIP values < 1 (**A-b**) indicate no significant correlation. (**B**) For the thymus index, only Calycosin–7–glucoside demonstrates both positive regression (**B-a**) and VIP > 1 (**B-b**), confirming its specific protective effect on the thymus. (**a**: regression coefficient, **b**: VIP value; 1: Calycosin–7–glucoside, 2: Ononin, 3: Calycosin, 4: Quercetin, 5: Genistein, 6: Isoliquiritigenin, 7: Formononetin, and 8: Medicarpin).

As shown in Figure 14, the regression coefficient of the A–a spleen index shows Ononin, Quercetin, and Calycosin, all of which were positively correlated with the spleen index, but in Figure 14A-b, the variable importance for the projection (VIP value) is less than 1, so there was no statistical significance. The regression coefficient of the B–a thymus index shows that Calycosin–7–glucoside, Ononin, Genistein, and Quercetin were positively correlated with the thymus index, but only Calycosin–7–glucoside had a VIP greater than 1, suggesting that it had a positive protective effect on the thymus.

#### 2.4.2. Correlation Analysis Between Gastrointestinal–Related Factors and Main Components

**Figure 15:**
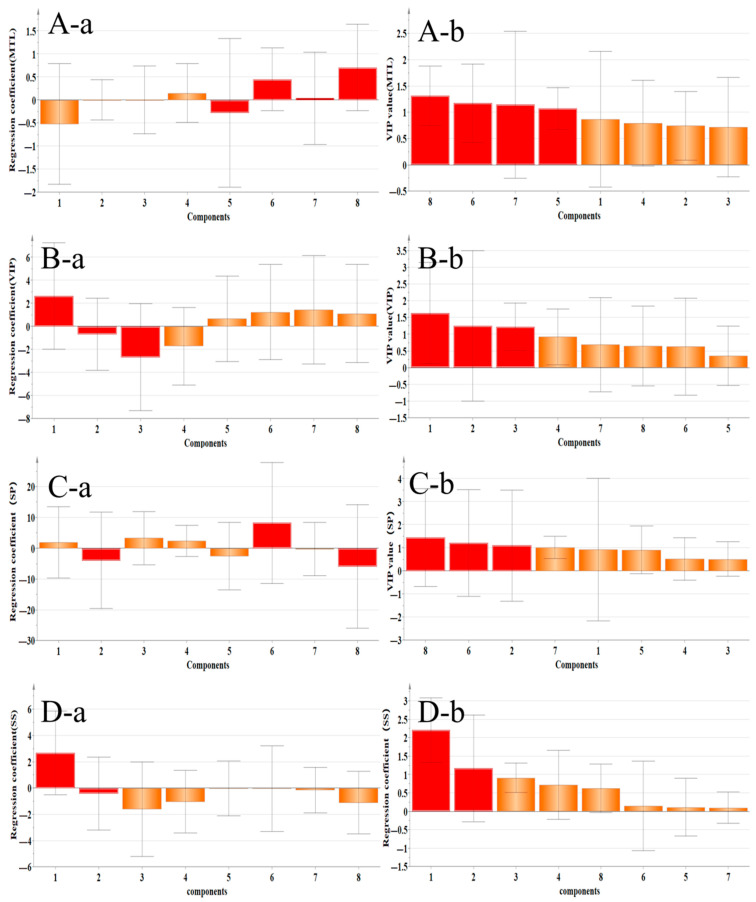
Correlation analysis between flavonoid components and gastrointestinal regulatory factors. (**A**) Medipterin, Isoliquiritigenin, and Formononetin (VIP > 1) show significant positive correlations with MTL secretion, suggesting their role in gastric motility regulation. (**B**) Only Calycosin–7–glucoside (VIP > 1) demonstrates validated association with VIP levels, indicating specific protection via intestinal peptide modulation. (**C**) Isoliquiritigenin (VIP > 1) exhibits the strongest correlation with substance P (SP), implicating neurokinin-mediated mucosal repair. (**D**) Calycosin-7-glucoside (VIP > 1) solely associates with somatostatin (SS) secretion, reflecting gastric acid secretion regulation. (**a**: regression coefficient, **b**: VIP value; 1: Calycosin–7–glucoside, 2: Ononin, 3: Calycosin, 4: Quercetin, 5: Genistein, 6: Isoliquiritigenin, 7: Formononetin, and 8: Medicarpin).

Figure 15 shows the correlation regression coefficient diagram and variable importance diagram of gastrointestinal–related factors and components. The components that were positively related to MTL secretion were Medipterin, Isoliquiritigenin, Formononetin, and Quercetin, and the VIP values of the first three components were greater than 1, which suggested that these three components can protect the gastrointestinal tract through the MTL factor, and Medipterin was better. The components positively related to the secretion of VIP were Calycosin–7–glucoside, Isoliquiritigenin, Formononetin, and Genistein, and the VIP value of Calycoside was greater than 1, which indicated that this component had a positive protective effect on the gastrointestinal tract through the VIP factor, and the effect was stronger. Isoliquiritigenin, Calycosin, Calycosin–7–glycoside, and Quercetin were positively correlated with SP secretion, and the VIP value of Isoliquiritigenin was greater than 1, which indicated that this component had a positive protective effect on the gastrointestinal tract through the SP factor. The component that was positively related to the secretion of SS was Calycosin–7–glycoside, and the VIP value of Calycosin–7–glycoside was greater than 1, which indicated that this component had a positive protective effect on the gastrointestinal tract through the SS factor.

#### 2.4.3. Correlation Analysis Between Immune Factors and Main Components

**Figure 16:**
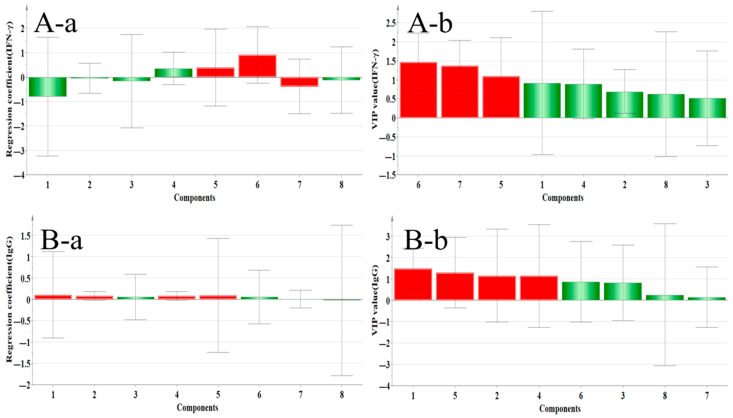
Correlation analysis between flavonoid components and immune factors (IFN–γ/IgG). (**A**) Isoliquiritigenin and Genistein (VIP > 1) show significant positive correlations with IFN–γ secretion, indicating their immunomodulatory effects through Th1 response. (**B**) Calycosin–7–glucoside demonstrates the strongest IgG correlation (VIP > 1), suggesting its role as the primary component enhancing humoral immunity. (**a**: regression coefficient, **b**: VIP value; 1: Calycosin–7–glucoside, 2: Ononin, 3: Calycosin, 4: Quercetin, 5: Genistein, 6: Isoliquiritigenin, 7: Formononetin, and 8: Medicarpin).

Figure 16 shows the regression coefficient diagram and variable importance diagram of the correlation between immune factors and main components. The secretion of IFN–γ was positively correlated with Quercetin, Genistein, and Isoliquiritigenin, but only Isoliquiritigenin and Genistein VIP values were greater than 1, suggesting that these two components were positively correlated with the secretion of IFN–γ, which can have systemic immune effect through IFN–γ factor, and Isoliquiritigenin is better; B–a shows that the components positively related to IgG secretion were Calycosin–7–glucoside, Ononin, Calycoside, Genistein, Quercetin, and Isoliquiritigenin, and the VIP values of the first four components were greater than 1, suggesting that these components could regulate immunity through the IgG factor, and Calycosin–7–glucoside was the best.

#### 2.4.4. Correlation Analysis Between Inflammatory Factors and Main Active Components

**Figure 17:**
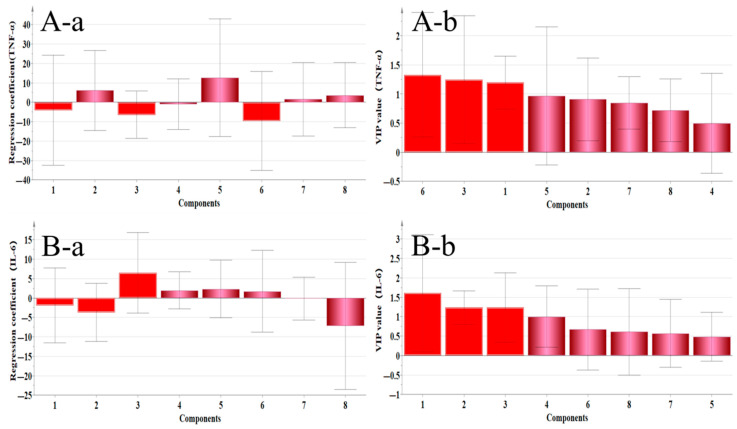
Correlation analysis between flavonoid components and inflammatory factors (IL–6/TNF–α). (**A**) Calycosin (VIP > 1) shows significant positive correlation with IL–6 levels, suggesting its role in inflammation regulation, while Isoliquiritigenin, Quercetin, and Genistein show weaker associations. (**B**) No components met the VIP > 1 threshold for TNF–α correlation, indicating that TNF–α modulation requires alternative pathways. (**a**: regression coefficient, **b**: VIP value; 1: Calycosin–7–glucoside, 2: Ononin, 3: Calycosin, 4: Quercetin, 5: Genistein, 6: Isoliquiritigenin, 7: Formononetin, and 8: Medicarpin).

Figure 17 shows a correlation analysis diagram of inflammatory factors and components. A–a shows that the components positively related to IL–6 secretion were Calycosin, Isoliquiritigenin, Quercetin, and Genistein, and the VIP value of Calycosin was greater than 1, suggesting that this component could improve inflammation through IL–6 factor; B–a shows that Ononin, Genistein, Formononetin, and Medipterin were positively correlated with the secretion of TNF–α, but their VIP values were less than 1, so there was no statistical significance.

## 3. Discussion

The results of content determination showed that among the four extracts based on the water extract, n–BuOH and EtOAc had better enrichment on the eight components. Among them, n–BuOH extract showed higher enrichment efficiency for Calycosin–7–glucoside and Ononin, while the EtOAc extract was more effective for Calycosin–7–glucoside, Ononin, and Calycosin, with higher contents of each component. The content of Ononin reached 22.2778 μg/g in EtOAc. These findings suggest that the extraction sites of n–BuOH and EtOAc may be the main active sites of RH, and Ononin and Calycosin–7–glucoside may be the key components of RH.

Although traditional Chinese medicine offers advantages of multi–targets and multi–pathways, its complex composition makes comprehensive pharmacological studies using animal models challenging. In view of this, we used the method of network pharmacology analysis to predict and analyze the target pathway. The results show that the key components may be Quercetin, Calycosin, Formononetin, Naringenin, and Calycosin–7–glucoside, and IL6, HSP90AA1, MAPK3, MAPK1, ESR1, IL1B, IFNG, RELA, RAF1, and CHUK were the key targets. GO and KEGG analyses revealed that the Biological Processes involved in the genes of RH against gastrointestinal injury caused by chemotherapy were cellular response to chemical stress, molecular biological functions, nuclear receptor activity, ligand–activated transcription factor activity, receptor ligand activity, etc. It is also associated with the Pathways in cancer, the IL–17 signaling pathway and the TNF signaling pathway.

General behavioral assessments revealed that rats in the MCG exhibited progressive declines in food intake, water consumption, body weight, and organ indices compared to the BCG, confirming successful establishment of the chemotherapy–induced gastrointestinal injury model. In contrast, these parameters significantly increased in the PCG relative to the MCG, validating the therapeutic efficacy of the reference drug. The n–BuOH group exhibited a slight decrease compared with other drug groups in body weight, indicating a notable protective effect against gastrointestinal injury. Both the n–BuOH and the PE groups were found to promote spleen growth in rats, and the n–BuOH and the RWG significantly enhanced thymus growth. Collectively, these behavioral data suggested that n–BuOH demonstrated the most pronounced protective effect against damage to gastrointestinal and immune system organsimmune organs resulting from chemotherapy.

Gastrointestinal motility was regulated by gastrointestinal hormones, which will secrete abnormally after being poisoned by chemotherapy drugs, thus leading to gastrointestinal dysfunction. The network pharmacology analysis showed that the protective effect of RH on gastrointestinal injury caused by chemotherapy was related to target proteins such as IL–6, IL1B, IFNG, and RELA. In this study, serum factors associated with gastrointestinal inflammation and immunity were selected as detection indices. Vasoactive Intestinal Peptide (VIP) is a brain–gut peptide released by intestinal neurons that can relax gastrointestinal smooth muscle [5]. Motilin (MTL) is an excitatory gastrointestinal hormone that promotes gastric contraction and segmental movement of the small intestine, accelerates intestinal transit time, and increases colon motility [5]. An increase in the expression levels of VIP and MTL in gastrointestinal tissues could lead to disordered gastrointestinal motility and secretion functions, resulting in abdominal pain and diarrhea [5]. Substance P (SP) has the highest concentration in the gastrointestinal tract, which can contract gastrointestinal smooth muscle and stimulate intestinal mucosa to secrete electrolytes [5,19]. Somatostatin (SS) [20] can inhibit the secretion and release of various gastrointestinal hormones [21], and the content of SS increases after gastrointestinal injury [22]. Interleukin–6 (IL–6) is a common pro–inflammatory factor that plays a significant role in the inflammatory cascade and mediates cellular immunity. Tumor necrosis factor–alpha (TNF–α), primarily produced by activated macrophages, exhibits anti–infective effects and is a critical inflammatory mediator. Reduced immune function could result in gastrointestinal dysfunction and inflammation, which may lead to atrophy and shedding of the gastrointestinal mucosa, ultimately decreasing the digestive and absorptive capacity of the gastrointestinal tract [23,24]. Interferon–gamma (IFN–γ), predominantly a pro–inflammatory cytokine produced by T cells and natural killer cells, triggers immune regulatory responses through multiple signaling pathways [24]. Ig is the most important immune molecule in humoral immune response, and Immunoglobulin G (IgG) is the most important antibody in human serum. Our research indicates that utilizing Zhenqi Fuzheng Granules as the positive control drug was a reasonable choice, with each administration group demonstrating a positive regulatory effect. Notably, n–BuOH exhibited the most significant effect in reducing MTL and SP levels; EtOAc was most effective in lowering VIP content; HPS showed the greatest reduction in SS levels; and n–BuOH again proved most effective for MTL and SP. Additionally, IL–6 and IFN–γ were the most effective in reducing their respective contents, while CHCl_3_ was particularly effective in lowering IgG levels, and HPS was most effective in reducing TNF–α levels. By decreasing the levels of these factors, inflammation can be mitigated, thereby enhancing the body’s immunity and exerting a protective effect on the gastrointestinal tract. Overall, n–BuOH and HPS demonstrated superior recovery levels of serum factors. These two agents were utilized for the staining of gastrointestinal tissue in the drug group and for pathological tissue observation. The results of this part of the test showed that compared with the BCG, the gastrointestinal pathology of the MCG rats was significantly different, with the mucosal structure destroyed and bleeding more severe. For n–BuOH, HPS, and CHCl_3_, compared with the MCG, the pathological manifestations were reduced to varying degrees, and the improvement in n–BuOH was more obvious.

Network pharmacology analysis indicated that the primary pathway through which RH mitigated gastrointestinal injury induced by chemotherapy was the “Pathways in Cancer”. Notably, the pathway included the interaction between cytokines and their receptors. The upstream cytokine, KITLG, interacted with the cytokine receptor KIT. Subsequently, the PI3K–Akt signaling pathway activated IKK, leading to the phosphorylation of IκBα. Then, in conjunction with NF–κB, it targets downstream effectors, ultimately promoting the evasion of apoptosis, reducing cell death, and providing a protective effect on the organism. Consequently, we focused our pathway research on the upstream stem cell factor receptor protein c–kit and its ligand SCF, as well as the downstream protein NF–κB p65 within the “Pathways in Cancer”. Additionally, our observations of the general behavioral status of rats, along with pathological tissue examination and serum factor detection, revealed that n–BuOH and HPS exhibit significant pharmacological effects. Therefore, these two parts were selected for Western Blot (WB) analysis to validate the pathway through which RH counters chemotherapy–induced gastrointestinal injury. The SCF/c–kit signaling pathway represented a novel direction in the study of gastrointestinal diseases. Tyrosine kinase receptor (c–kit), a transmembrane protein encoded by the proto–oncogene c–kit, exhibits tyrosine kinase activity [25]. The stem cell factor (SCF) was a glycoprotein [26], and gastric antral SCF was produced by smooth muscle cells in the gastric wall, whose effects were mediated through the c–kit receptor. In the rat gastrointestinal tract, Cajal interstitial cells (ICCs) had the capacity to regulate gastrointestinal motility. The morphology, structure, and quantity of ICCs directly influence gastrointestinal motility and are crucial for normal peristalsis [27,28]. c–kit serves as a specific receptor for ICCs, primarily secreted by smooth muscle cells and activated by SCF [29]. Upon SCF binding to the c–kit receptor in ICCs, a dimer complex was formed, which initiated downstream signaling pathways, such as MAPK and Ras. This activation ultimately engaged intracellular transcription factors, regulating gene expression and controlling ICC cell growth, proliferation, and development, thereby enhancing gastrointestinal motility and potentially exacerbating diarrhea [30]. Additionally, the nuclear factor–kB (NF–κB) pathway was implicated in gastric injury, as well as protection and repair processes [31]. Its activation or overexpression can worsen gastric mucosal damage. Research has indicated that the activation of the NF–κB signaling pathway may significantly contribute to the overexpression of pro–inflammatory genes in the gastric mucosa, particularly in a gastric ulcer model involving stress in rats [32]. Our research results showed that the n–BuOH part of RH could protect the stomach and intestines by down–regulating the expressions of SCF, c–kit, and p65 proteins, which was the same in Xie’s [33] study. HPS played a protective role against gastric injury by down–regulating the expression of c–kit and p65 proteins and played a protective role in the intestinal injury by down–regulating the expressions of SCF and c–kit protein. This indicated that RH protected the gastrointestinal tract by regulating the expression of SCF/c–kit/p65 protein.

The correlation analysis employed a VIP value threshold of greater than 1 to comprehensively evaluate components that exhibit positive correlations. The regression coefficient indicated the contribution of each component to gastrointestinal protection and a larger regression coefficient signified a higher contribution rate. Regarding immune organs, Calycosin–7–glucoside demonstrated a positive protective effect on the thymus. In terms of gastrointestinal health, Calycosin–7–glucoside, Medicarpin, and Isoliquiritigenin were positively correlated with gastrointestinal protection, with Calycosin–7–glucoside exhibiting the strongest effect. Concerning immune factors, both Isoliquiritigenin and Calycosin–7–glucoside were positively associated with immune regulation. In relation to inflammatory factors, Calycosin showed a positive correlation with inflammation regulation. Based on above analysis, we found that Calycosin–7–glucoside emerged as the most significant component of RH, demonstrating a positive protective effect against gastrointestinal injury induced by chemotherapy, which appeared to follow a certain “dose–effect” relationship.

Our results demonstrate that the n–BuOH fraction of RH significantly attenuated cisplatin–induced gastrointestinal injury, consistent with prior reports on RH’s gastroprotective effects in ulcerative colitis models [9]. The down–regulation of SCF/c–kit/NF–κB pathway proteins aligns with known mechanisms of flavonoid–mediated mucosal protection, while the superior efficacy of Calycosin–7–glucoside corroborates its established role as a key bioactive compound in Hedysarum species [13,34,35]. Notably, our correlation analysis extends previous findings by establishing dose–effect relationships between specific flavonoids and gastrointestinal recovery markers, advancing the pharmacological understanding of RH’s multi–target action.

While this study provides valuable insights into RH’s protective mechanisms against chemotherapy–induced gastrointestinal injury, certain aspects merit further exploration. The current approach, focusing on correlating individual flavonoid constituents with bioactivity through PLS–DA, does not fully address potential inter–component interactions within the active n–butanol fraction. Future investigations employing combinatorial methodologies such as fraction reconstitution or isobolographic analysis could help elucidate whether the observed efficacy derives principally from Calycosin–7–glucoside or emerges from flavonoid synergies [36,37]. Additionally, while the acute cisplatin rodent model offers relevant pharmacological insights, it may not fully capture the clinical complexity of multi–drug, multi–cycle chemotherapy regimens in human patients. The translation of therapeutically effective doses to clinically applicable regimens [38], along with evaluation of potential interactions between RH preparations and diverse chemotherapeutics, represents an important direction for subsequent research

## 4. Materials and Methods

### 4.1. Materials and Reagents

Radix Hedysari was purchased from Ruida Chinese Herbal Medicine Professional Cooperative (Longnan City, China). It was identified by Professor Zhijia Cui (Gansu University of Chinese Medicine) as the dried root of Hedysarum polybotrys Hand. –Mazz. (Fabaceae), verifying compliance with the standards specified in the Chinese Pharmacopoeia. Cisplatin for injection (freeze–dried) (No. 1J0535B03) came from Qilu Pharmaceutical Co., Ltd. (Jinan, China). Zhenqi Fuzheng Granules (No. K20210842) were purchased from Gansu Fuzheng Pharmaceutical Technology Co., Ltd. (Dingxi, China). The 4% paraformaldehyde universal tissue fixative was bought from Guangzhou Saiguo Biotechnology Co., Ltd. (Guangzhou, China). The 0.9% sodium chloride injection was purchased from Chenxing Pharmaceutical Co., Ltd. (Shandong, China). ELISA kits for MTL, SP, SS, VIP, TNF–α, IL–1β, IL–6, IgM, IgG, and IFN–γ were purchased from RUIXIN BIOTECH (Quanzhou, China). The BCA protein concentration assay kit (No. P0010) was from Beyotime Biotechnology (Shanghai, China). SDS (No. 30166428), glycerol (No. 10010618), methanol (No. 10014118), NaCl (No. 10016318), and KCl (No. 10020318) were purchased from Sinopharm Chemical Reagents Co., Ltd. (Shanghai China). The 14–120KD protein marker (No. DM111) was purchased from Beijing Quanshijin Biotechnology Co., Ltd. (Beijing, China). The 10–250KD protein marker (No. 26619–1) was purchased from fermentas (Shenzhen, China). The 0.45 μm PVDF membrane (No. IPVH00010) and 0.22 μm PVDF membrane (No. ISEQ15150) were purchased from Millipore. SCF (No. 26582–1–AP), NF–κB (No. 66535–1–Ig), Gapdh (No. 60004–1–Ig), and c–kit (No. 60004–1–Ig) were purchased from proteintech (Wuhan, China). Hematoxylin stain, eosin stain, differentiation solution, and neutral gum were purchased from Shanxi Yike Biotechnology Service Co., Ltd. (Xian, China). Anhydrous ethanol and xylene were purchased from Sinopharm Group Chemical Reagent Co., Ltd. (Shanghai, China). Chromatographic–grade methanol (No. 20210515), acetonitrile (No. 20220103), analytical–grade 95% ethanol (No. 20220106), ethyl acetate (No. 20210201), petroleum ether (60~90 °C, No. 20210104), chloroform, N–butanol, formic acid (No. 20201101), and chloral hydrate (No. 20210504) was purchased from Tianjin Damao Chemical Reagent Factory (Tianjin, China). Calycosin–7–glucoside (Y16O11H127829), Ononin (M02A11S120167), Calycosin (Y29J12H139162), Quercetin (C28J11Y116820), Genistein (H30A9Z69019), Isoliquiritigenin (H03D9Z76567), Formononetin (H06S9Z69494), and Medicarpin (Y12M11H112983) were all purchased from Shanghai Yuanye Biotechnology Co., Ltd. (Shanghai, China).

### 4.2. Preparation of Different Extracts

Two portions of RH (500 g each) were extracted twice under reflux with boiling water and 95% ethanol (sample: solvent, 1: 10, *w*/*v*) for 2.0 h and once with 95% ethanol (sample: solvent, 1:10, *w*/*v*) for 1.5 h. The filtrates from each solvent were collected separately. The 95% ethanol extract was concentrated under reduced pressure until no residual alcohol odor was detected. The aqueous extract was dried to constant weight. This yielded the water extract (WE) and the 95% ethanol extract (95% EtOH) of RH.

Furthermore, we weighed 800 g of *RH* medicinal material to create a water extract using the aforementioned extraction method. The resulting water extract was concentrated to 150 mL and then sequentially extracted with equal volumes of petroleum ether, chloroform, ethyl acetate, and n–butanol. This was shaken and mixed thoroughly, allowing the mixture to stand for 15 min before extracting each layer separately until the color remained unchanged. The solution from each extraction was combined, enriched, and freeze–dried to obtain the petroleum ether (PE), chloroform (CHCl_3_), ethyl acetate (EtOAc), n–butanol (n–BuOH), and residual water (RWE) extraction parts. The remaining liquid was mixed with anhydrous ethanol until the alcohol content reached 77%. We allowed the mixture to undergo ethanol precipitation for 10 h to obtain a brown, sticky precipitate. The precipitate was then dissolved in ten times the amount of hot water and centrifuged for 20 min (25 °C, 3000 r/min) while the solution was still hot. After the supernatant was cooled, absolute ethanol was added until the alcohol content reached 77%, and the precipitate was deposited for 10 h. The alcohol precipitate was once again dissolved in ten times its volume of hot water, and a third alcohol precipitation was performed in the same method. The precipitate was filtered using suction and rinsed with ether, acetone, and absolute ethanol to yield an off–white powder, identified as the crude polysaccharide of Hedysarum hedysari (HPS). The yields of different extraction parts are shown in Table 5.

### 4.3. HPLC Quantification of Flavonoids

#### 4.3.1. HPLC Conditions

**Figure 18:**
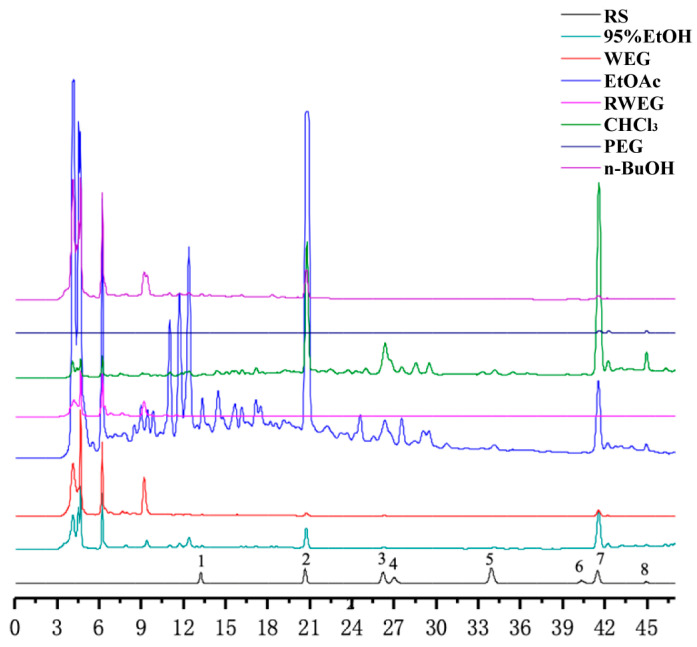
HPLC chromatogram of eight flavonoids in different polar parts (1: Calycosin–7–glucoside, 2: Ononin 3: Calycosin, 4: Quercetin, 5: Genistein, 6: Isoliquiritigenin, 7: Formononetin, and 8: Medicarpin. RS: reference substance, 95% EtOH: 95% ethanol extract, WE: water extract, EtOAc: ethyl acetate, RWE: residual water extract, CHCl_3_: chloroform, PE: petroleum ether, and n–BuOH: n–butanol).

The Waters e2695 high–performance liquid chromatography (HPLC) system and standard reference substance were used for quantitative and qualitative analyses of 8 flavonoids (Figure 1) in 7 polar parts of RH. The chromatographic column was SinoChrom ODS–AP (250 mm × 4.6 mm, 5 μm), and the temperature was 25 °C. Mobile phase A was acetonitrile, mobile phase B consisted of 0.2% formic acid in water, and the flow rate was set at 0.6 mL/min. The gradient program was used as follows: 0~15 min, 17~33% A; 15~20 min, 33~34% A; 20~24 min, 34~37% A; 24~27 min, 37~38% A; 27~30 min, 38~40% A; 30~35 min, 40~43% A; and 35~45 min, 43~90% A. The detection wavelength was set at 260 nm, and the injection volume was 10 μL. Under the conditions of this chromatography, both the experimental sample and the control sample were analyzed. The chromatogram is shown in Figure 18.

#### 4.3.2. Preparation of Reference Substance and Sample Solution

Medicarpin, Ononin, Isoliquiritigenin, Calycosin–7–glucoside, Genistein, Calycosin, Quercetin, and Formononetin reference substances were weighted accurately, and we added methanol to prepare a mixed reference substance solution containing 0.2400, 0.2880, 0.2400, 0.2400, 0.2520, 0.2400, 0.2640, and 0.2760 mg per 1 mL, respectively. The water extract, the 95% ethanol extract, the ethyl acetate extract, the chloroform extract, the petroleum ether extract, the n–butanol extract, and the residual water part were weighed precisely to 0.2500 g of each, dissolved in 5 mL with methanol, and filtered to obtain the final product.

### 4.4. Prediction of “Target–Protein–Pathway” of RH Against Gastrointestinal Injury Induced by Chemotherapy [39,40]

#### 4.4.1. Network Pharmacology Workflow

The online database Traditional Chinese Medicine Systems Pharmacology (TCMSP) was utilized to screen the chemical constituents of RH. To enhance the representativeness of the screened active components, thresholds of oral bioavailability (OB) ≥ 30% [31] and drug likeness (DL) ≥ 0.18 were established during the screening process. Based on the identified compounds, corresponding predicted protein targets were obtained from the TCMSP platform. Also, the Canonical SMILES formula of the compound can be obtained on the online platform Pubchem, then the target of the compound can be predicted on the online platform Swiss Target Prediction, and then all target names can be corrected to gene names by using the online database Uniprot. To identify disease targets related to gastrointestinal injury caused by chemotherapy, the search term “Gastrointestinal injury caused by chemotherapy” was employed across the three databases: Genecards, CTD, and OMIM. The Draw Venn Diagram platform was used to map the three databases, the intersection target was selected to construct the disease target database, then the intersection target with the obtained active ingredient target was selected, and finally the potential target of RH in protecting gastrointestinal injury caused by chemotherapy was screened out.

#### 4.4.2. Construction of “Drug–Compound–Target” Network

The identified potential targets in 2.4.1 were imported into Cytoscape 3.10.2 software to create a network diagram, which was used to explore the pharmacological mechanisms of RH for protecting against chemotherapy–induced gastrointestinal injury.

#### 4.4.3. Construction of PPI Protein Interaction Network and Screening of Key Targets

The potential targets obtained in 2.4.1 were introduced into the String platform to construct a protein interaction network, with a primary interaction network established by selecting a confidence threshold of 0.900. Key targets were determined through topological analysis using Cytoscape 3.10.2 software.

#### 4.4.4. GO and KEGG Analysis

The common targets identified in 2.4.1 were then input into the David database for Gene ID conversion to the Ensemble Gene ID. Finally, the resulting file was imported into the Omicshare platform and micro–information platform [41], where the species was set to Homo sapiens, with a *p* value threshold of <0.01, to conduct visual analyses of KEGG and GO.

### 4.5. Screening of Effective Fractions and Study on Mechanism of Action [42]

#### 4.5.1. Animals

Male and female Wistar rats weighing 180~220 g were purchased from the Experimental Animal Center of Lanzhou Veterinary Research Institute of the Chinese Academy of Agricultural Sciences (license number: SCXR (Gan) 2020–0002; quality certificate number: 002370, Lanzhou, China). All animals were maintained under SPF standard laboratory conditions (temperature 22~25 °C, humidity 55~65%, and dark/light for 12 h cycles). The animals had free access to food and distilled water. All experimental procedures were approved by the Experimental Animal Ethics Committee of Gansu University of Chinese Medicine (Lanzhou, China). The certificate number of ethics examination was 2021–259, and the license number of laboratory animal facilities was SYXK (Gan) 2020–0009.

After approximately one week of adaptive feeding, rats were numbered sequentially with picric acid. They were randomly divided into 11 groups with 10 rats in each group. These groups were named the blank control group (BCG), model control group (MCG), positive control group (PCG), water extract group (WEG), 95% ethanol extract group (95% EtOH), petroleum ether extract group (PE), chloroform extract group (CHCl_3_), ethyl acetate extract group (EtOAc), n–butanol extract group (n–BuOH), Hedysarum polysaccharide group (HPS), and residual water group (RWEG).

With the exception of the BCG, all rats were intraperitoneally injected with 1 mg/kg of cisplatin for 7 consecutive days to establish the models. The BCG and the MCG were administered 0.2 mL of 0.9% sodium chloride solution by gavage for 7 consecutive days; the positive control group received Zhenqi Fuzheng Granules at a dosage of 0.54 g/kg by gavage for the same duration. At the same time, intragastric administration was performed. The drug group was also administered 0.2 mL of 0.9% sodium chloride solution by gavage for 7 consecutive days. Additionally, oral administration was given to the water extract group, 95% ethanol extract group, petroleum ether extract group, chloroform extract group, ethyl acetate extract group, n–butanol extract group, polysaccharide group, and residual water part group at a dosage of 2.7 g/kg, calculated according to the amount of crude drug [18].

#### 4.5.2. Monitoring of General Behavior

Starting from day one, each rat was administered a daily ration of 250 g of food and 500 mL of water. On the following day, each rat was weighed, and the remaining food and water were measured to monitor food intake, water consumption, and weight changes. After the spleen and thymus were dissected and washed with normal saline, the body mass was weighed, and the thymus index and spleen index were calculated.

*Organ index* = *Organ mass/body mass*

#### 4.5.3. Collection of Serum and Specimens

On the sixth day after administration, food was forbidden but water continued for 18 h. On the 7th day, rats were anesthetized by intraperitoneal injection of 5% chloral hydrate after 1 h of being modeled, gavaged, and given drugs. Twenty minutes later, blood was collected from the abdominal aorta, allowed to sit at room temperature for 2 h, and then centrifuged at 4 °C at 3500 r/min for 15 min. The serum was separated using a centrifugal radius of 6.2 cm and stored in a −80 °C freezer for later use. Additionally, after the blood collection, the gastric antrum and ileum, spleen, and thymus were immediately dissected, washed with physiological saline, and divided into equal portions. Half of the samples were preserved in 4% paraformaldehyde, while the other half were rapidly frozen in liquid nitrogen and stored in a −80 °C freezer for future experiments.

#### 4.5.4. Detection of Serum Factor

The frozen serum samples from each group were transferred from a −80 °C freezer to a 4 °C refrigerator for rethawing and subsequently allowed to equilibrate at room temperature for 30 min prior to use. The enzyme–linked immunosorbent assay (ELISA) was employed to quantify the levels of Motilin (MTL), Vasoactive Intestinal Peptide (VIP), Substance P (SP), Somatostatin (SS), Interferon Gamma (IFN–γ), as well as Immunoglobulin G (IgG), Interleukin 6 (IL–6), and Tumor Necrosis Factor α (TNF–α).

#### 4.5.5. Pathological Detection by H&E Staining

The fresh tissues of the rat stomach and intestine were fixed with 4% paraformaldehyde solution for more than 24 h; then, the tissues were leveled and dehydrated with ethanol and xylene, and the dehydrated tissues were embedded in an embedding frame with melted paraffin. The embedded tissues were cooled in a freezing table at −20 °C, sliced in a microtome, flattened with warm water at 40 °C in a spreading machine, and baked in an oven at 60 °C for later use. Then, they were dyed via hematoxylin–eosin staining step by step and sealed with neutral gum, afterward being observed and analyzed under a microscope.

#### 4.5.6. Western Blot Analysis

We took the tissue protein solution that had been prepared by pretreatment and used BSA as the standard to calculate the regression equation and calculate the protein concentration of the sample. The protein was denatured using a metal bath and centrifuged, and the total protein was separated by 10% SDS–PAGE and transferred to a PVDF membrane.

After being sealed with 5% milk/TBST at a shaking table at room temperature for 1 h, the primary antibody was diluted with 1% BSA/PBST (1:1000), the membrane was sealed, and it was kept in the refrigerator at 4 °C overnight. The primary antibodies such as SCF, NF–κB, c–kit, and GAPDH were combined with the target protein, and then the goat anti–mouse secondary antibody (1:5000) labeled with horseradish peroxidase diluted with 5% milk/PBST was detected. Enhanced Luminol Reagent and Oxidizing Reagent were used to develop the color of the PVDF membrane, and the results were observed using a gel imaging system.

### 4.6. Correlation Analysis of “Dose–Effect”

In this experiment, the statistical software SIMCA–P13.0 was used to perform partial least square regression analysis with PLS–DA as the model [43]. The regression coefficient of eight flavonoids in RH under “4.3” was taken as the independent variable X, the organ index under “4.5.2” and the serum factor content under “4.5.5” were taken as the dependent variable Y, and the correlation between them was analyzed; then, the relationship between gastrointestinal injury and the main active components was preliminarily explained.

## 5. Conclusions

**Figure 19:**
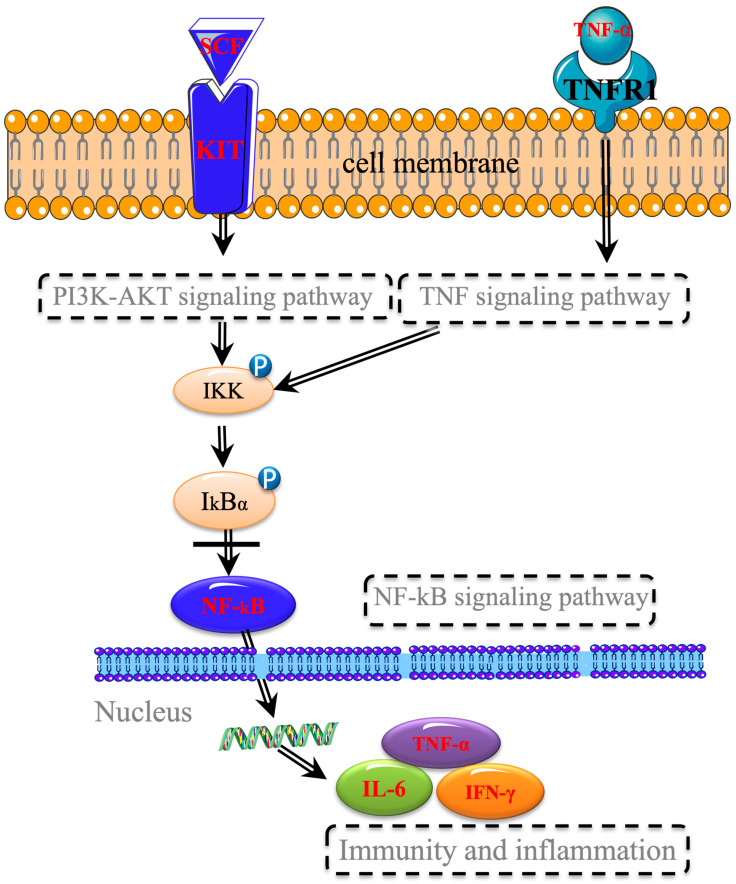
Mechanistic model of Radix Hedysari (RH) protection against cisplatin–induced gastrointestinal injury. (Color coding: Red nodes: Experimentally validated targets (SCF, c–kit, NF–κB p65, IL–6, TNF–α, IFN–γ). Black nodes: Intermediate regulators (TNFR1, IKK, IkBα). RH down–regulates SCF/c–kit signaling, blocking IκBα phosphorylation and NF–κB nuclear translocation, thereby: Suppressing pro–inflammatory cytokine production (IL–6↓, TNF–α↓), Restoring immune homeostasis (IFN–γ, IgG), Normalizing gastrointestinal motility factors (MTL, VIP, SP, SS)).

Our research demonstrated that the effective part of RH in mitigating gastrointestinal injury induced by chemotherapy was n–BuOH, with Calycosin–7–glucosid serving as its material basis, which exhibited a specific “dose–effect” relationship. Additionally, RH had the capacity to regulate associated serum factors, reduce inflammation, and enhance immunity via the c–kit/SCF/NF–κB signaling pathway. This provided a foundational basis for the clinical application of RH, as illustrated in Figure 19. RH down–regulated the expression of the stem cell factor (SCF) and its receptor c–Kit, subsequently inhibiting NF–κB activation and nuclear translocation. This molecular cascade led to reduced inflammatory cytokine production (IL–6 and TNF–α), improved immune regulation (IFN–γ and IgG), and normalized gastrointestinal motility factors (MTL, VIP, SP, and SS), ultimately protecting gastrointestinal mucosal integrity and function against cisplatin–induced damage. The diagram systematically integrates pharmacological actions at the molecular, cellular, and physiological levels, demonstrating RH’s multi–target therapeutic mechanism.

## Figures and Tables

**Table 1:** The content of each component in different polar parts (μg/g).

	WE	95% EtOH	EtOAc	CHCl_3_	PE	n–BuOH	RWE
Calycosin–7–glucoside	3.0149	2.3015	9.1728	0.1366	0.0128	6.8075	0.0965
Ononin	1.6171	2.8848	22.2778	1.2574	0.1193	6.5518	0.1669
Calycosin	1.2201	1.2348	7.8777	2.0956	0.0100	0.9642	0.0930
Quercetin	0.4948	0.6551	8.0639	0.6411	0.0088	0.8874	——
Genistein	0.4624	0.8606	0.7293	0.1659	——	0.2242	0.5757
Isoliquiritigenin	0.8077	1.0296	0.8593	0.2022	——	——	——
Formononetin	0.6932	1.1765	0.6604	0.3986	0.0136	0.2949	0.2626
Medicarpin	0.5689	1.5039	1.6939	0.7397	0.3220	0.4956	0.2055
Total content	8.8791	11.6468	51.3351	5.6371	0.4865	16.2256	1.4002

**Table 2:** PPI core target information.

Target Name	Protein	Degree	Closeness Centrality	Betweenness Centrality
IL6	Interleukin 6	20	0.613	0.88
HSP90AA1	Heat shock protein HSP 90–alpha	19	0.49	0.84
MAPK3	MAP kinase–activated protein kinase 3	17	0.77	0.53
MAPK1	Mitogen–activated protein kinase 1	17	0.75	0.64
ESR1	Estrogen receptor	17	0.55	0.23
IL1B	Interleukin–1 beta	15	0.49	0.30
IFNG	Interferon gamma	12	0.47	0.05
RELA	Transcription factor p65	11	1.00	0.09
RAF1	RAF proto–oncogene serine	8	0.60	0.14
CHUK	Inhibitor of nuclear factor kappa–B kinase subunit alpha	7	0.40	0.06

**Table 3:** Organ index of thymus and spleen.

Group	Spleen Index	Thymus Index
BCG	2.59 ± 0.35 ^a^	2.15 ± 0.69 ^a^
MCG	2.14 ± 0.32 ^bc^	1.25 ± 0.64 ^bc^
PCG	2.31 ± 0.24 ^ab^	1.65 ± 0.50 ^b^
WEG	2.12 ± 0.22 ^bc^	1.10 ± 0.32 ^c^
95% EtOH	2.16 ± 0.23 ^bc^	1.18 ± 0.45 ^bc^
PE	2.27 ± 0.41 ^ab^	1.24 ± 0.47 ^bc^
CHCl_3_	2.26 ± 0.29 ^bc^	1.23 ± 0.37 ^bc^
EtOAc	2.18 ± 0.19 ^bc^	1.20 ± 0.41 ^bc^
n–BuOH	2.27 ± 0.31 ^ab^	1.42 ± 0.48 ^ab^
HPS	2.05 ± 0.22683 ^c^	1.27 ± 0.71 ^bc^
RWEG	2.23 ± 0.25 ^bc^	1.41 ± 0.36 ^ab^

(Note: The data in the same column have the same lowercase letters, indicating that there is no significant difference (*p* > 0.05); different lowercase letters indicate significant differences (*p* < 0.05)).

**Table 4:** Effects of RH extracts on spleen and thymus indices in cisplatin–treated rats (*n* = 10).

	Regression Coefficient > 0	VIP > 1	Positive Correlation Component
Spleen index	Ononin, Quercetin, and Calycosin	Genistein, Isoliquiritigenin, and Formononetin	No statistical significance
Thymus index	Calycosin–7–glucoside, Ononin, Genistein, and Quercetin	Isoliquiritigenin, Formononetin, and Calycosin–7–glucoside	Calycosin–7–glucoside
VIP	Calycosin–7–glucoside, Isoliquiritigenin, Medicarpin, Formononetin, and Isoliquiritigenin	Calycosin–7–glucoside, Ononin, and Calycosin	Calycosin–7–glucoside
SS	Calycosin–7–glucoside	Calycosin–7–glucoside and Ononin	Calycosin–7–glucoside
MTL	Medicarpin, Isoliquiritigenin, Formononetin, and Quercetin	Medicarpin, Isoliquiritigenin, Formononetin, and Genistein	Medicarpin, Isoliquiritigenin, and Formononetin
SP	Isoliquiritigenin, Calycosin, Calycosin–7–glucoside, and Quercetin	Ononin, Isoliquiritigenin, and Medicarpin	Isoliquiritigenin
IFN–γ	Quercetin, Genistein, and Isoliquiritigenin	Genistein, Isoliquiritigenin, and Formononetin	Isoliquiritigenin and Genistein
IgG	Calycosin–7–glucoside, Ononin, Calycosin, Genistein, Quercetin, and Isoliquiritigenin	Calycosin–7–glucoside, Ononin, Genistein, and Quercetin	Calycosin–7–glucoside, Ononin, Genistein, and Quercetin
TNF–α	Ononin, Genistein, Formononetin, and Medicarpin	Calycosin–7–glucoside, Ononin, and Calycosin	No statistical significance
IL–6	Calycosin, Isoliquiritigenin, Quercetin, and Genistein	Calycosin–7–glucoside, Ononin, and Calycosin	Calycosin

**Table 5:** Quality and yield of different extracts of RH.

Extract	Weight of Extract (g)	Yield (%)
WE	115.65	23.13
95% EtOH	76.30	15.26
PE	0.42	0.05
CHCl_3_	0.76	0.10
EtOAc	0.69	0.09
n–BuOH	19.9	2.49
RWE	23.30	2.91
HPS	3.94	0.49

## Data Availability

Data will be made available on request.

## Abbreviations

Radix Hedysari, RH; oral bioavailability, OB; drug likeness, DL; biological process: BP; cellular component, CC; molecular function, MF; enzyme–linked immunosorbent assay, ELISA; variable importance for the projection, VIP; Western blot, WB; hematoxylin–eosin staining, HE; high–performance liquid chromatography, HPLC; blank control group, BCG; model control group, MCG; positive control group, PCG; water extract group, WEG; 95% ethanol extract group, 95% EtOH; petroleum ether extract group, PE; chloroform extract group, CHCl_3_; ethyl acetate extract group, EtOAc; n–butanol extract group, n–BuOH; Hedysarum polysaccharide group, HPS; and residual water group, RWEG.
